# Risk Factors for Urosepsis after Minimally Invasive Percutaneous Nephrolithotomy in Patients with Preoperative Urinary Tract Infection

**DOI:** 10.1155/2020/1354672

**Published:** 2020-01-02

**Authors:** Shen Wang, Peng Yuan, Ejun Peng, Ding Xia, Hua Xu, Shaogang Wang, Zhangqun Ye, Zhiqiang Chen

**Affiliations:** Department of Urology, Tongji Hospital, Tongji Medical School, Huazhong University of Science and Technology, Wuhan 430030, China

## Abstract

The purpose of this study was to assess risk factors of urosepsis after minimally invasive percutaneous nephrolithotomy (MPCNL) for the treatment of upper urinary tract stones in patients with preoperative urinary tract infection (UTI) and to explore preventive measures. Between 2008 and 2016, patients with preoperative UTI who underwent MPCNL for upper urinary tract stones were retrospectively collected. Patients were divided into nonurosepsis and urosepsis groups. Perioperative outcomes of all patients were evaluated and compared between the two groups. Risk factors for post-MPCNL urosepsis were investigated using univariate and multivariate regression analysis. A total of 843 patients including 22 patients with postoperative urosepsis (urosepsis group) and 821 patients without urosepsis (nonurosepsis group) were finally included in this study. All patients with postoperative urosepsis were cured and discharged after treatment. In univariate analysis it was demonstrated that the incidence of urosepsis after MPCNL was significantly correlated with channel size (*P*=0.001), surgical time (*P*=0.003), as well as the tear of the collection system and percutaneous renal channel crossing the renal papilla (*P*=0.004). Moreover, multivariate analysis showed that smaller channel size (OR = 11.192, 95% CI: 2.425–51.650, *P*=0.002), longer surgical time (OR = 6.762, 95% CI: 1.712–17.844, *P*=0.008), and tear of collection system and percutaneous renal channel crossing the renal papilla (OR = 5.531, 95% CI 1.228–14.469, *P*=0.012) were independent risk factors for urosepsis following MPCNL in patients with preoperative UTI. In conclusion, in patients with preoperative UTI undergoing MPCNL for upper urinary tract stones, smaller channel size, prolonged operation time, as well as tear of the collection system and percutaneous renal channel crossing the renal papilla are independent risk factors for postoperative urosepsis. Therefore, it is indicated that, in clinical practice, it is of great significance to choose appropriate channel size and avoid renal injury and control surgical time to prevent the urosepsis after MPCNL in patients with preoperative UTI.

## 1. Introduction

Urinary stone disease is one of the most common ailments in urology surgery. Patients with urinary tract stones account for more than 50% of inpatients, with the ailment ranking first in urology surgery [1]. Clinically, stones of the upper urinary tract account for about 95% of all urinary stones. Minimally invasive surgical methods other than open surgeries were usually first considered on most common patients, among which percutaneous nephrolithotomy (PCNL) has become one of the preferred treatment options for upper urinary tract stones and it has been recommended that PCNL can be served as the first-line treatment of larger stones >2 cm [2, 3].

The development of minimally invasive technology promoted the application of a new procedure of minimally invasive percutaneous nephrolithotomy (MPCNL) in which both a miniature endoscope and a smaller access tract were used [4]. MPCNL has gradually drawn attention from urological surgeons for its superiority in reducing surgical complications such as hemorrhage, organ damage, and the loss of renal function compared with traditional open surgeries or standard PCNL [5, 6]. Moreover, previous studies have found no significant difference in stone-free rate between MPCNL and standard PCNL [7, 8]. Generally, the overall stone-free rate after MPCNL is considerable, which can be 85–95% [9]. MPCNL can be also served as a feasible method in patients with complex stones like staghorn calculi [10].

Urosepsis, as the most serious complication of infection, poses potential threats on patients after endoscopic therapy for the treatment of urinary tract stones. Urosepsis is defined as one kind of sepsis associated with urinary tract infection (UTI), which takes up 5% of all sepsis types [11]. The pathological process from UTI to urosepsis, which is sometimes rapidly evolved, may not be easily identified in clinical practice. However, upon occurrence, urosepsis with a high mortality rate seriously endangers patients' life [12]. Previous studies have found that there are several risk factors for urosepsis after PCNL, including preoperative, intraoperative, as well as postoperative factors. Main risk factors included positive urine culture, stone size, stone complexity, surgical time, renal pelvic pressure, and residual stones [13, 14].

It has been found that urosepsis after MPCNL might lead to high mortality especially among patients with complex stones [15]. It is of great importance for clinicians to detect postoperative systemic inflammatory response syndrome (SIRS) and prevent SIRS from progressing to urosepsis [16]. Previous studies have found a significant association between preoperative UTI and urosepsis after endoscopic surgeries for upper urinary tract stones [17, 18]. Although the management of antibiotic therapy or drain therapy may reduce the incidence of urosepsis, the potential risks of preoperative UTI remain unclear. In the current study, it is aimed to analyze risk factors for urosepsis after MPCNL for the treatment of upper urinary tract stones in patients with preoperative UTI, thereby comprehensively evaluating the safety of MPCNL and providing a theoretical basis for preventing and treating postoperative urosepsis in these patients.

## 2. Materials and Methods

Between January 2008 and December 2016, the clinical data of patients with preoperative UTI who underwent unilateral MPCNL with holmium-laser for the treatment of upper urinary tract stones in Tongji Hospital of Tongji Medical College, Huazhong University of Science and Technology, were retrospectively analyzed. Patients were included based on following criterions: (1) diagnosed with unilateral single upper urinary tract stone by urological ultrasound or Computed Tomography (CT); (2) with the American Society of Anesthesiologists (ASA) Grade I–III; (3) with preoperative UTI (diagnosed by significant syndromes, pyuria, and/or positive urine culture); (4) receiving MPCNL after hemogram and urine routine tests restored normal results only by antibiotic anti-infective treatment but without drainage treatment (ureteral stent or percutaneous nephrostomy); (5) with one channel size of <24F during PCNL; (6) without tumors, diabetes, anemia, chronic renal insufficiency, oral immunosuppressive agents, or renal congenital malformations, without the infection in other organs, and without previous renal surgery; (7) with all available data for the study.

### 2.1. Surgical Methods

All patients underwent MPCNL with holmium-laser under B-Ultrasonography guidance, routine gradual expansion for channel construction, and perfusion with an irrigation pump (flow rate: 400 mL/min). The specific procedure was as follows: after the patient was administered continuous epidural anesthesia, a 6F ureteral catheter was retrogradely indwelled in the renal pelvis or upper ureter. Then, the patient was placed in the prone position, and normal saline was injected into the ureteral catheter to induce artificial hydronephrosis. Subsequently, a puncture of the calyceal fornix was carried out under B-Ultrasonography guidance at the 11th intercostal region or 12th costal margin, between the posterior axillary and scapular lines. A successful puncture was reflected by the outflow of clear urine. Then, the guidewire was inserted into the renal pelvis or ureter and the needle sheath was pulled out. This was followed by dilation along the guidewire with a fascia dilator, starting from 12F and gradually continued with a step of F2 to construct the desired PCNL channel. Next, a ureteroscope or a nephroscope was inserted, followed by holmium-laser lithotripsy and stone removal.

If the constructed percutaneous renal channel directly entered the pelvis through the renal papilla, this was referred to as a case of percutaneous renal channel crossing the renal papilla. When the renal collection system showed a tear, a case of tear of the renal collection system was considered. After no residual stones were detected by B-ultrasonography, double J and nephrostomy tubes were conventionally indwelled.

Stone composition was analyzed by an automatic stone infrared spectrum analysis system (Lanmode Scientific Instrument Co., Ltd. Tianjin, China). At postoperative 1 week, the patients were assessed by the plain film or urinary CT scan to determine the presence or absence of residual stones (diameter of ≥3 mm) as well as whether there was a need for adjuvant therapy.

### 2.2. Outcome Measures

Baseline and perioperative outcomes were collected and evaluated in all patients. In the present study, the renal injury was defined as a tear of the collection system and percutaneous renal channel crossing the renal papilla. Moreover, urosepsis was diagnosed according to Guidelines for Management of Severe Sepsis and Septic Shock (2016 version) cosigned by the Society of Critical Care Medicine (SCCM), the European Society of Intensive Care Medicine (ESICM), and the International Sepsis Forum (ISF) [3].

### 2.3. Statistical Analysis

Statistical analyses were performed with the SPSS 23.0 software. Continuous and discrete variables were, respectively, described as mean ± standard deviation (range from minimum to maximum), and *N* (%). Risk factors were assessed by univariate analysis, with normally distributed data analyzed by *t*-test and Pearson's *X*^2^ test; nonnormally distributed data were assessed by Mann–Whitney *U* test and Spearman's correlation test. Multivariate logistic regression analysis was used to determine risk factors for postoperative urosepsis. A difference with *P* < 0.05 was considered statistically significant.

## 3. Results

**Figure 1:**
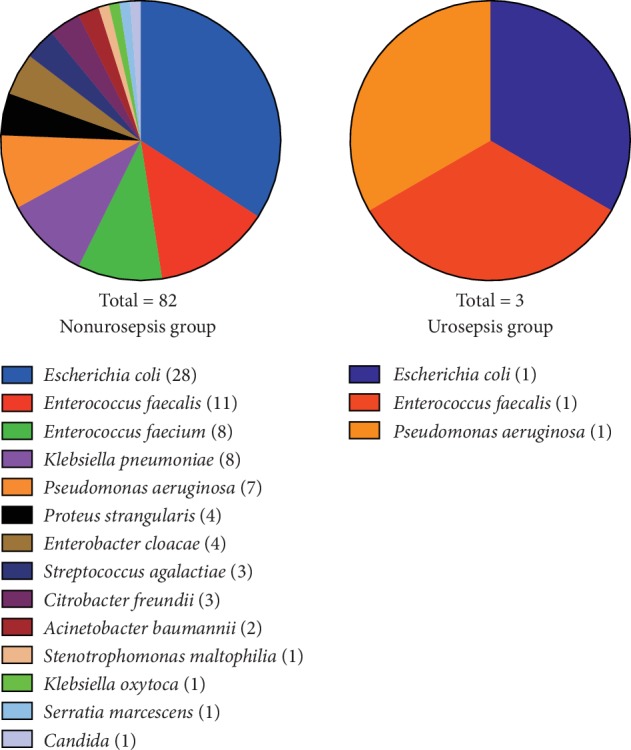
Microbiological results of preoperative urine culture in all patients of the two groups. No significant difference in the microorganism species of preoperative positive urine culture was observed between the two groups (*P*=0.946).

**Figure 2:**
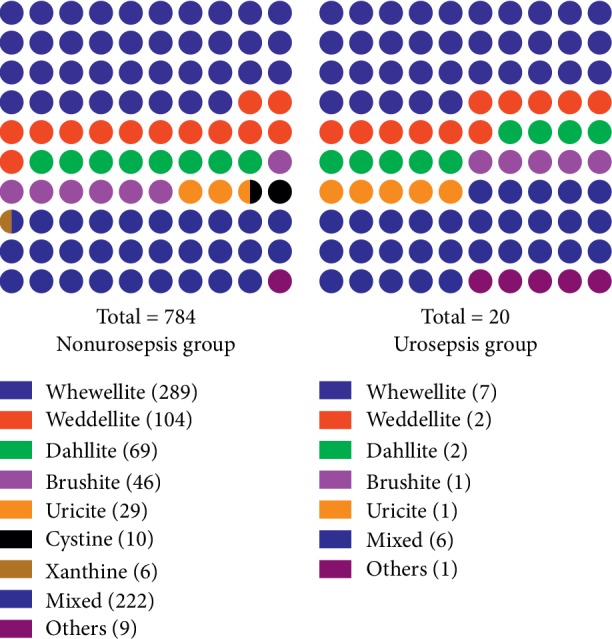
The results of noninfectious stone composition in all patients of the two groups. There was no significant difference in the types of noninfectious stones between the two groups (*P*=0.930).

A total of 843 patients, including 821 patients without urosepsis after MPCNL (nonurosepsis group) and 22 patients who suffered from urosepsis postoperatively (urosepsis group), were finally included in this study. The baseline characteristics of all patients in the two groups were shown in Table 1. Preoperative urine culture was positive in 82 patients (nonurosepsis group) and 3 patients (urosepsis group). Microbiological results of preoperative urine culture were displayed in Figure 1. It has been found that Gram-negative bacteria and *Escherichia coli* were most common in both groups. Moreover, there was no significant difference in the microorganism species of preoperative urine culture between the two groups (*P*=0.946). The results of noninfectious stone composition were presented in Figure 2. And it was demonstrated that there was no significant difference in the types of noninfectious stones between the two groups (*P*=0.930).

All patients received the third generation cephalosporins (*n* = 521, 61.8%) or quinolone (*n* = 322, 38.2%) for treating preoperative UTI as the experiential therapy until the drug sensitivity result was confirmed. According to the drug sensitivity analysis, the third generation cephalosporins (especially the third generation cefoperazone combined with sulbactam) had been most recommended.

Among 22 patients in the urosepsis group, results of postoperative vital signs, white blood cell (WBC), platelet, serum procalcitonin (PCT), C-reactive protein (CRP), as well as urine and blood culture were shown in Table 2. These data had been collected at the time of patients' initial diagnosis of urosepsis after the operation. And all patients with postoperative urosepsis were cured after active treatments and all patients in the study were discharged with good recovery.

Baseline characteristics and other clinical parameters were included in the univariate analysis. Univariate analysis showed that smaller channel size (OR = 5.209, 95% CI: 2.016–13.459, *P*=0.001), prolonged operation time (OR = 4.951, 95% CI: 1.589–15.312, *P*=0.003), and tear of the collective system and percutaneous renal channel crossing the renal papilla (OR = 4.579, 95% CI: 1.477–14.193, *P*=0.004) were associated with the occurrence of urosepsis. Meanwhile, factors including age, sex, BMI, stone side, stone location, stone diameter, the extent of hydronephrosis, stone composition, preoperative urine culture, and presence of residual stones were not significantly related to the occurrence of urosepsis (Table 3).

Multivariate regression analysis revealed that smaller channel size (OR = 11.192, 95% CI: 2.425–51.650, *P*=0.002), longer operation time (OR = 6.762, 95% CI: 1.712–17.844, *P*=0.008), and tear of the collective system and percutaneous renal channel crossing the renal papilla (OR = 5.531, 95% CI 1.228–14.469, *P*=0.012) were significant risk factors for the occurrence of urosepsis (Table 3).

**Figure 3:**
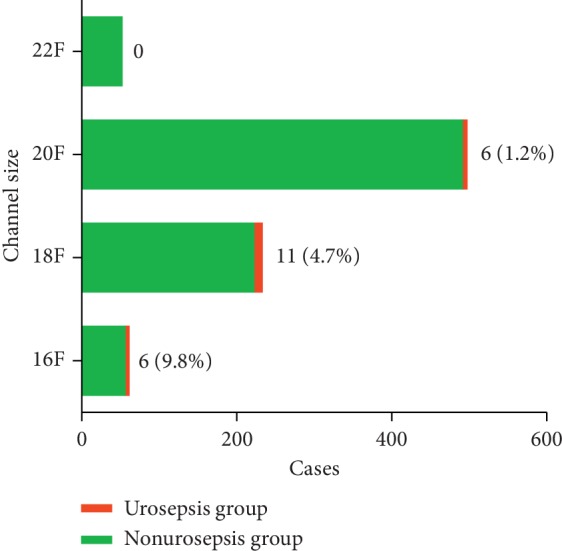
Relationship between channel size of MPCNL and the incidence of urosepsis. The cases of urosepsis were shown as number (%) on the right of the bar. Statistical analysis showed that *P* value was ≤0.001.

Finally, the patients were subgrouped according to the channel size. 16F channel was used in 61 cases, with the urosepsis incidence of 8.2%. 18F channel was employed in 233 cases with the urosepsis incidence of 4.7%. 20F channel was used in 497 cases with the urosepsis incidence of 1.2%. 22F channel was utilized in 52 cases with no sepsis cases. It was indicated that the incidence of urosepsis was significantly correlated with channel size (*P* ≤ 0.001) (Figure 3).

## 4. Discussion

Clinically, the main reasons for urosepsis are the following: (1) MPCNL has been performed on patients with UTI before the infection is fully controlled; (2) the possibility of live pathogenic bacteria in infectious stones; (3) complex renal stones, with heavy loads and a long operation time; (4) excessively high intrarenal pressure intraoperatively [19–23]. In this study, we focused on patients who underwent MPCNL with preoperative UTI for a more sensitive and effective observation of the impacts of surgical factors on sepsis, aiming to reduce sepsis incidence by improving surgical procedures and methods. Nevertheless, 22 patients still suffered from postoperative urosepsis.

Univariate and multivariate analyses demonstrated that the smaller the surgical channel, the higher the probability of sepsis. It has been known that in terms of physics, in case of constant renal pelvis perfusion flow within a time unit, the smaller the outflow channel (percutaneous renal channel), the higher the intrapelvic pressure. A large number of studies have found that the mechanism of sepsis after MPCNL mainly includes two aspects: urinary tract infection and an excessively high intrapelvic pressure, which cause bacteria and associated toxins to enter the blood stream via the renal pelvis vein and the lymph, resulting in urosepsis [24, 25]. Additionally, a study comparing PCNL via a minimally invasive technology with a standard channel demonstrated that for complex renal and infectious stones, the larger the surgical channel, the lower the sepsis incidence [26–28]. Larger channels could be implemented in patients with potential UTI or a large stone load, in order to reduce the renal pelvic pressure and prevent sepsis. Thus, corroborating the above findings, MPCNL should be recommended in such patients after full consideration and a standard channel may be preferred.

Moreover, it was found that operative time was also an independent risk factor for urosepsis. The longer the operative time, the higher the risk of sepsis. Previous studies have also revealed that the risk of infection is significantly increased with a surgical time longer than 90 min during PCNL [17, 19]. Operative time mainly depends on stone loads and channel size. Larger load and smaller operation channels may result in a longer operative time and a higher probability of infection. Therefore, we suggest that staging operation should be an alternative in patients with stone overload, rather than excessively pursuing the stone-free rate at the first attempt if operation time is predicted to be long and the risk of infection is expected to be high.

In addition, the mucosa in the renal collection system, which has a protective effect, can prevent urinary bacteria and toxins from entering the blood stream. The incidence of urosepsis was significantly increased when the collection system was torn or the percutaneous renal channel crossed the renal papilla rather than the calyceal fornix. It is likely that blood vessels are damaged with the puncture channel crossing the renal papilla or the tear of renal papilla due to excessive tilting of the ureteroscope, which may make bacteria and toxins enter the blood circulation and cause sepsis. Hence, the calyceal fornix should be punctured accurately and the operation should be performed gently during MPCNL, while tilting the ureteroscope in a large angle should be avoided.

It is indicated that the following measures should be adopted for patients with preoperative UTI who are to receive MPCNL: (1) administration of antibiotics to control UTI preoperatively; (2) use of a large channel intraoperatively, maintaining a low perfusion flow, ensuring smooth drainage of perfusion fluid, and reducing renal pelvis pressure; (3) shortening of operation time; (4) making sure that calyceal fornix puncture is achieved, avoiding intraoperative tear of the collection system and mucosal damage.

On the other hand, the key for urosepsis treatment is early detection and timely intervention, including (1) maintaining the drainage patency of the nephrostomy tube; (2) quickly supplementing 2000–3000 ml crystalloid and colloid solution and monitoring central venous pressure; (3) selecting effective antibiotics according to susceptibility test results; (4) for adequate fluid infusion, it is recommended to apply norepinephrine as soon as possible to improve the hemodynamic status and protect renal function; (5) applying a large-dose of glucocorticoids in the early stage when necessary.

There are some limitations in the current study. The retrospective nature of the study and the limited number of patients may be disadvantageous, and the results of renal pelvic pressure were not available in the study. However, it is difficult to carry out the measurement of renal pelvic pressure in most primary hospitals. So the relationship between channel size and the incidence of urosepsis was investigated and discussed in this study. More well-designed prospective studies with a large sample are demanded in future studies.

## 5. Conclusions

In conclusion, it was found that channel size, operation time, and tear of the collection system and percutaneous renal channel crossing the renal papilla were independent risk factors for postoperative urosepsis after MPCNL for the treatment of upper urinary tract stones in patients with preoperative UTI, and early detection and effective preventions are necessary for such patients.

## Figures and Tables

**Table 1:** Baseline characteristics of all patients in the study.

	Nonurosepsis group (*n* = 821)	Urosepsis group (*n* = 22)	*P*
Age (years)	46.2 ± 20.5 (17–80)	47.3 ± 18.5 (16–82)	0.342

Sex			0.872
Male	471 (57.4%)	13 (59.1%)	
Female	350 (42.6%)	9 (40.9%)	

BMI (kg/m^2^)	24.3 ± 2.6 (17.5–34.7)	25.1 ± 3.4 (18.1–33.6)	0.714

Stone side			0.529
Left	392 (47.7%)	12 (54.5%)	
Right	429 (52.3%)	10 (45.5%)	

Stone type			0.650
Upper ureteral stones	50 (6.1%)	1 (4.6%)	
Complex renal stones	128 (15.6%)	5 (22.7%)	
Other renal stones	643 (78.3%)	16 (72.7%)	

Stone diameter (cm)	2.8 ± 1.4 (1.6–3.9)	3.3 ± 1.8 (1.5–3.8)	0.449

Extent of hydronephrosis			0.536
≤2 cm	276 (33.6%)	5 (22.7%)	
>2, <4 cm	433 (52.7%)	13 (59.1%)	
≥4 cm	112 (13.6%)	4 (18.2%)	

Preoperative urine culture			0.479
Positive	82	3	
Negative	739	19	

Stone composition			0.312
Infectious stone (Struvite)	37	2	
Noninfectious stone	784	20	

BMI: body mass index.

**Table 2:** Postoperative results of 22 patients in urosepsis group.

	Urosepsis group (*n* = 22)
T (°C), *n*; <36/36–38/>38	3/3/16

P (bpm), *n*; </>90	1/21

R (bpm), *n*; </>20	3/19

Urine WBC, *n*; +∼++/+++∼	8/14

Abnormal blood WBC, *n*; <4/>10 × 10^9^/L	2/18

Blood WBC (×10^9^/L)	14.2 ± 6.9 (1.6–33.4)

Abnormal blood platelet, *n*; <100/>300 × 10^9^/L	4/1

Blood platelet (×10^9^/L)	149.0 ± 59.4 (86–356)

Abnormal serum PCT^*∗*^, *n *(%)	20 (90.9%)

Serum PCT (ng/ml)	9.5 ± 6.7 (0.32–21.2)

Abnormal serum CRP^*∗∗*^, *n *(%)	19 (86.3%)

Serum CRP (mg/L)	96.9 ± 51.8 (4.3–189)

Positive urine culture, *n* (%)	8 (36.4%)
Pseudomonas aeruginosa	2 (9.1%)
Klebsiella pneumoniae	2 (4.5%)
Escherichia coli	1 (4.5%)
Acinetobacter baumannii	1 (4.5%)
Streptococcus agalactiae	1 (4.5%)
Enterococcus faecalis	1 (4.5%)

Positive blood culture, *n* (%)	2 (9.1%)
Escherichia coli	1 (4.5%)
Pseudomonas aeruginosa	1 (4.5%)

T: temperature; P: pulse; R: respiration; WBC: white blood cell; PCT: procalcitonin; CRP: C-reactive protein; ^*∗*^<0.5 ng/ml; ^*∗∗*^<10 mg/L.

**Table 3:** Univariate and multivariate analysis results in the study.

	Nonurosepsis group (*n* = 821)	Urosepsis group (*n* = 22)	Univariate analysis		Multivariate analysis	
			OR (95% CI)	*P*	OR (95% CI)	*P*
Age, >/≤65 years	214/607	5/17	0.834 (0.304–2.289)	0.725		
Sex, M/F	471/350	13/9	1.073 (0.454–2.539)	0.872		
BMI, >/≤28 kg/m^2^	263/558	6/16	1.257 (0.486–3.249)	0.636		
Stone side, L/R	392/429	12/10	0.761 (0.325–1.782)	0.529		
Complex/common stone	128/693	5/17	1.592 (0.577–4.393)	0.365		
Stone diameter, >/≤3 cm	247/574	8/14	1.328 (0.550–3.206)	0.527		
Infectious/other stone	37/784	2/20	0.472 (0.106–2.095)	0.312		
Preoperative urine culture positive/negative	82/739	3/19	1.423 (0.412–4.912)	0.479		
Hydronephrosis, >/≤4 cm	739/82	18/4	2.003 (0.662–6.060)	0.210		
Single/multiple channel	729/92	17/5	2.331 (0.840–6.466)	0.095		
Channel size, ≤/>18F	278/543	16/6	5.209 (2.016–13.459)	**0.001**	11.192 (2.425–51.650)	**0.002**
Operation time (mins)	47.58 ± 8.25	65.68 ± 6.19	4.951 (1.589–15.312)	**0.003**	6.762 (1.712–17.844)	**0.008**
Renal injury	38 (4.6%)	4 (18.2%)	4.579 (1.477–14.193)	**0.004**	5.531 (1.228–14.469)	**0.012**
Residual stones	87 (10.6%)	2 (9.1%)	1.185 (0.272–5.157)	0.821		

M: male; F: female; L: left; R: right; BMI: body mass index; OR: odds ratio; CI: confidence interval.

## Data Availability

The data used to support the findings of this study are available from the corresponding author upon request.
